# Sociocultural and masculinity influences on colorectal cancer screening participation among Hispanic/Latino men in Florida, New York, and Texas

**DOI:** 10.1002/cam4.70159

**Published:** 2024-09-20

**Authors:** Ami Elizabeth Sedani, Jessica Yasmine Islam, Derek M. Griffith, Kelly Krupa Rifelj, Cordero McCall, Omar García‐Rodríguez, Marlene Camacho‐Rivera, Charles Ray Rogers

**Affiliations:** ^1^ Division of Epidemiology & Social Sciences, Institute for Health & Equity Medical College of Wisconsin Milwaukee Wisconsin USA; ^2^ Center for Immunization and Infections in Cancer, Cancer Epidemiology Program H. Lee Moffitt Cancer and Research Institute Tampa Florida USA; ^3^ Center for Men's Health Equity, Racial Justice Institute Georgetown University Washington Columbia USA; ^4^ School of Nursing University of Pennsylvania Philadelphia Pennsylvania USA; ^5^ Department of Community Health Sciences SUNY Downstate Health Sciences University Brooklyn New York USA

**Keywords:** colorectal neoplasms, early detection of cancer, Hispanic or Latino, masculinity, men's health

## Abstract

**Background:**

This cross‐sectional study explored how masculinity beliefs may influence colorectal cancer (CRC) screening participation among ethnic subgroups of screening‐age–eligible (45–75 years) Hispanic/Latino men.

**Methods:**

Using a consumer panel, we recruited self‐identified Hispanic/Latino men fluent in English or Spanish, and residing in Florida, New York, or Texas. The Masculinity Barriers to Medical Care (MBMC) scale and its six subscales were used to assess masculinity beliefs. Multivariable logistic regression was used to estimate the association between MBMC and CRC screening participation, adjusting for Hispanic/Latino subgroup, marital status, survey language, age group, and health insurance status. Results were then stratified by Hispanic/Latino subgroup.

**Results:**

Of the participants (n=611), approximately 31% identified as Puerto Rican, 30% as other Hispanic/Latino, 26% as Mexican, and 14% as Cuban; 63% had ever been screened for CRC. We found no differences in the prevalence of screening participation by Hispanic/Latino subgroup. The majority of participants had completed both a stool‐based test and an exam‐based screening test (29.3%). After adjusting for confounding, MBMC reduced the odds of screening participation. Slight MBMC‐subscale differences were observed by Hispanic/Latino subgroup. For example, higher scores on the *Restrictive Emotionality* subscale were associated with a lower likelihood of screening participation among Puerto Rican men, but higher odds of screening for Cuban men.

**Conclusions:**

Masculinity barriers to CRC screening may exist. Tailored interventions to address masculinity barriers among specific Latino subgroups may improve CRC screening uptake in this population.

## Graphical abstract

In this cross‐sectional study involving 611 self‐identified Hispanic/Latino men in Florida, New York, and Texas, we examined differences in colorectal cancer (CRC) screening participation by Ethnic subgroup, and the influence of masculinity beliefs on CRC screening participation. There were no significant differences in screening participation by Ethnic subgroups. Overall, higher Masculinity Barriers to Medical Care (MBMC) scale scores were associated with reduced odds of screening participation, with specific MBMC subscale effects varying by subgroup.

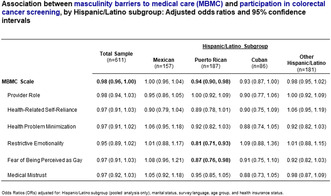

## 1 INTRODUCTION

Colorectal cancer (CRC) is the second leading cause of cancer death in the United States (US). Early‐detection screening for CRC is underutilized despite evidence that it significantly reduces CRC mortality.^1^, ^2^, ^3^ Notably, disparities in CRC mortality and morbidity across various racial/ethnic groups are far‐reaching; additionally, more men than women die from CRC each year in the United States.^4^, ^5^ Uptake of CRC screening is low among US Hispanic/Latinx populations; for example, only 61.7% of Hispanic/Latino men are up‐to‐date with screening compared with 74.6% of non‐Hispanic/Latino (NHL) white men.^6^, ^7^ Consequently, Hispanic/Latino men are more likely than NHL white men to be diagnosed with advanced CRC, even after accounting for differences in age and socioeconomic status, setting the stage for poor outcomes.^6^, ^8^ Recent data from the National Cancer Institute's Surveillance, Epidemiology, and End Results program show that, from 2012 to 2019, while the rate of new CRC cases across all racial and ethnic groups fell by 0.9% each year, the rate of new cases in the Hispanic/Latinx population did not significantly change.^9^ Variations in CRC‐related inequities among Hispanic/Latino men may be poorly described as, to date, most outcome data for this ethnic group are reported in aggregate.^10^, ^11^

The Hispanic/Latinx population is the largest minority group in the United States, comprising more than 20 distinct heritage subgroups.^12^ Hispanic identity among those residing in the United States is complex and diverse, shaped by various factors including politics, self‐perception, and geography. Over time, individuals of Latino heritage have adopted different labels to identify themselves. This identification can vary across immigrant generations, with “Hispanic” commonly referring to individuals from Spanish‐speaking countries and “Latino” typically used for those from Latin America. The presentation in aggregate of data and outcomes for the Hispanic/Latinx community as a whole may be masking important inequities in health outcomes among subgroups within this population.^11^, ^12^, ^13^, ^14^ For example, a study of Texas residents found that CRC mortality rates in US‐born Hispanic men surpassed those in Non‐Hispanic (NH) white men (24.1 vs. 18.1 per 100,000, respectively).^13^ Thus, disaggregating potential barriers to CRC screening within this diverse population is essential. Similar to other marginalized communities in the United States, Hispanic/Latino individuals may experience increased vulnerability to cancer disparities due to higher poverty rates, a greater proportion lacking health insurance coverage, limited transportation options, lack of awareness, and additional barriers to accessing quality healthcare, such as limited English proficiency.^14^, ^15^, ^16^, ^17^ The paucity of research examining heterogeneity in CRC screening among Hispanic/Latino men suggests that a potentially important opportunity to reduce inequities in CRC mortality is being overlooked. Moreover, further examination of screening rates by the diverse identities and characteristics within this community could reveal additional disparities embedded within Hispanic/Latinx subgroups.^18^

An understudied area among Hispanic/Latino men in the context of CRC screening is the psychosocial impacts of masculinity on health and how barriers due to masculinity beliefs influence screening participation rates.^19^, ^20^, ^21^, ^22^, ^23^, ^24^, ^25^, ^26^ The psychosocial impacts of masculinity, often associated with machismo, can impede Hispanic/Latino men's access to preventive care.^27^ Cultural norms among Hispanic/Latino men may discourage seeking healthcare—CRC screening include, as it could be seen as a sign of weakness, while traditional health beliefs emphasize stoicism and self‐reliance.^28^, ^29^ Prior research in other racial/ethnic groups of men has shown that endorsement of traditional masculinity and gender norms is associated with decreased participation in preventive health behaviors and with negative attitudes toward asking for help or toward behaviors perceived as indicating vulnerability.^30^, ^31^, ^32^ This exploratory study aimed to examine the association between masculinity beliefs and CRC screening participation in a sample of ethnically diverse Hispanic/Latino men in three states. Insights into the determinants of CRC screening by Hispanic/Latino ethnicity may facilitate tailored interventions appropriate for different subpopulations.

## 2 METHODS

### 2.1 Data source and study sample

This cross‐sectional study was approved by the Institutional Review Board (IRB) at the University of Utah (IRB #00113679) prior to data collection. From February to March 2022, we partnered with Qualtrics (Qualtrics International, Inc., Provo, UT), to recruit a diverse convenience sample of Hispanic/Latino men. Briefly, the Qualtrics network of participant pools (the Marketplace) consists of sources such as targeted email lists, customer‐loyalty web portals, and social media, and has been shown to be a valid method of recruitment for populations that tend be difficult to reach or involve in research.^33^, ^34^, ^35^ Interested participants were directed to the online study cover letter and were required to provide their consent by selecting “yes” before proceeding to answer a set of eligibility questions. Eligible individuals included: (a) self‐identified as male; (b) were aged 18–75 years at the time of the study; (c) self‐described as Hispanic/Latino; and (d) resided in Florida, Texas, or New York, which are among the top five US states with the largest Hispanic/Latinx populations.^36^ The survey contained 94 items (including seven eligibility questions) and took up to 15 min to complete. Those who completed the survey were invited to choose a method of compensation (e.g., frequent flyer miles, points toward retail purchases).

To reach our target sample size of 500 participants per state (*N* = 1500), we implemented nonproportional quota sampling across four age categories (18–29; 30–44; 45–59; 60–75). Participants who met the eligibility criteria and provided consent were included in the study. For the current study, the sample was restricted to men within the age range (45–75 years) eligible for CRC screening (for those at average risk) as recommended by the US Preventive Services Task Force (USPSTF) in 2021.^37^ Respondents who did not pass Qualtrics's quality checks (i.e., those with a survey duration of one‐third or less of the median, known as speeding checks) were excluded from the analysis.

### 2.2 Measures

#### 2.2.1 Masculinity beliefs

To examine the association between masculinity beliefs and CRC screening history, we used the Masculinity Barriers to Medical Care (MBMC) scale, which is described elsewhere and has been validated by Rogers et al. among African American, White, and Indigenous men.^38^ The MBMC scale consists of six theoretically derived factors (i.e., subscales): (1) Provider Role; (2) Health‐related Self‐Reliance; (3) Health Problem Minimization; (4) Restrictive Emotionality; (5) Fear of Being Perceived as Gay; and (6) Medical Mistrust. For all subscales, individual items were assessed on a 5‐point Likert‐type scale ranging from 1 (Not at all true) to 5 (Completely true) (Figure S1). Ten questions were reverse coded. Higher scores suggest greater masculinity barriers to medical care. Prior research has shown this operationalization to be a reasonable method for assessing masculinity beliefs among diverse male populations.^39^

#### 2.2.2 Outcome: Colorectal cancer screening completion history

For screening history, participants were asked if they had ever completed an at‐home blood stool‐based test or ever had an exam‐based test (sigmoidoscopy or colonoscopy). Consistent with national surveys as well as previous studies, sigmoidoscopy and colonoscopy rates were measured and reported as a combined measure.^22^ If respondents answered yes to either or both questions, they were categorized as having a self‐reported history of CRC screening participation.

#### 2.2.3 Covariates

Covariates selected for this study were informed by the literature and included sociodemographic characteristics such as Hispanic/Latino subgroup, survey language (proxy for dominant language/language preference and subsequent acculturation), age group, geographical residence (state), sexual orientation, marital status, employment status, educational attainment, annual household income, health insurance status, having a regular medical care provider, having seen a provider in the past 12 months, being a current tobacco smoker, family history of cancer, and family history of CRC.^16^, ^40^

Consistent with the 2010 US Census Hispanic Origin question, and the American Community Survey (ACS) questionnaire, participants were asked if they are of Hispanic, Latino/a, or Spanish origin where one or more categories may be selected.^41^ Individuals were classified based on their responses, regardless of how they responded to the race question. Subgroups which reflect the largest Hispanic/Latino subgroups in the United States included: (1) Mexican (Mexican, Mexican American, Chicano); (2) Puerto Rican; (3) Cuban; and (4) Other Hispanic/Latino (i.e., other than Mexican, Mexican American, Chicano, Puerto Rican, or Cuban), which reflect the largest Hispanic/Latino subgroups in the U.S.^36^

### 2.3 Statistical analysis

Due to limited missing data for our covariates of interest (less than 2%), we used a complete‐case approach. We used descriptive statistics (frequency and percentage) to describe sample characteristics. We examined differences in sociodemographic characteristics of the study population overall and across Hispanic/Latino subgroups. We conducted chi‐square tests to evaluate the statistical significance of subgroup differences.

Next, we assessed differences in masculinity barriers within the study population overall and across Hispanic/Latino subgroups. For each participant, we calculated an overall MBMC score and a total score for each MBMC subscale. For each subscale, we ran a series of multivariable logistic regression models to assess the odds of CRC screening participation. To explore differences by Hispanic/Latino subgroup, we stratified results by subgroup. We employed a manual stepwise selection approach in combination with subject‐matter knowledge and data‐source limitations. We assessed collinearity among covariates and excluded highly correlated variables from the model to avoid multicollinearity. For instance, variables such as employment status and age, and insurance status, having a regular medical care provider, and having seen a medical care provider in the past 12 months, were likely to explain similar variability in the outcome. We adjusted each model for Hispanic/Latino subgroup (except the stratified models), marital status, survey language, age, and health insurance status. Adjusted odds ratios (aORs) with 95% confidence intervals (95% CI) were reported. All analyses were conducted in SAS 9.4 (SAS Institute, Cary, NC, USA). A two‐sided *p*‐value of <0.05 was considered statistically significant. Due to the nature of this analysis, we did not include an adjustment for multiple comparisons.

## 3 RESULTS

### 3.1 Sample characteristics

A total of 611 survey respondents were included in our analytical sample; respondents' sociodemographic characteristics by subgroup are shown in Table 1. Among participants that specified other Hispanic, Latino, or Spanish origin (*n* = 41), responses included: Spanish, South America, Central American, Dominican, Afro‐Latino, and multiracial (data not shown). Most respondents self‐identified as Puerto Rican, followed by other Hispanic/Latino, Mexican, and then Cuban. More than 40% resided in Florida, with the remainder split almost equally between New York and Texas. Most men completed the survey in English, were aged 45–59 years, were currently married, identified as straight/heterosexual, were currently employed, had health insurance, had a regular medical care provider, had seen a medical care provider within the past 12 months, and were not current tobacco smokers. CRC screening participation history for the study population was roughly 62.7%, with the majority of those respondents having ever completed both an exam‐based test and a stool‐based test.

**TABLE 1:** Sociodemographic characteristics of study sample, by subgroup (*N* = 611).

	Total (*n* = 611)	Mexican (*n* = 157 [25.7%])	Puerto Rican (*n* = 187 [30.6%])	Cuban (*n* = 86 [14.1%])	Other Hispanic/Latino (*n* = 181 [29.6%])	*p* Value
Survey language						
English	461 (75.5)	132 (84.1)	166 (88.8)	57 (66.3)	106 (58.6)	<0.0001*
Spanish	150 (24.6)	25 (15.9)	21 (11.2)	29 (33.7)	75 (41.4)	
Age group (years)						
45–59	388 (63.5)	112 (71.3)	126 (67.4)	36 (41.9)	114 (63.0)	<0.0001*
60–75	223 (36.5)	45 (28.7)	61 (32.6)	50 (58.1)	67 (37.0)	
Residence: State						
New York	177 (29.0)	12 (7.6)	106 (56.7)	6 (7.0)	53 (29.3)	<0.0001*
Texas	175 (28.6)	131 (83.4)	8 (4.3)	3 (3.5)	33 (18.2)	
Florida	259 (42.4)	14 (8.9)	73 (39.0)	77 (89.5)	95 (52.5)	
Marital status						
Married	325 (53.2)	84 (53.5)	89 (47.6)	49 (57.0)	103 (56.9)	0.5891
Divorced, widowed, or separated	169 (27.7)	45 (28.7)	58 (31.0)	23 (26.7)	43 (23.8)	
Never married	117 (19.2)	28 (17.8)	40 (21.4)	14 (16.3)	35 (19.3)	
Sexual orientation						
Straight/heterosexual	554 (90.8)	133 (84.7)	173 (92.5)	76 (88.4)	172 (95.0)	0.0044*
Nonheterosexual identity	57 (9.2)	24 (15.3)	14 (7.5)	10 (11.6)	9 (5.0)	
Educational attainment						
HS/GED or below	157 (25.7)	53 (33.8)	57 (30.5)	16 (18.6)	31 (17.1)	0.0001*
Some college	223 (36.5)	54 (34.4)	77 (41.2)	27 (31.4)	65 (35.9)	
Bachelor's degree or higher	231 (37.8)	50 (31.9)	53 (28.3)	43 (50.0)	85 (47.0)	
Household income						
Less than $15,000	54 (8.8)	10 (6.4)	23 (12.3)	10 (11.6)	11 (6.1)	0.2025
$15,000–$24,999	69 (11.3)	20 (12.7)	25 (13.4)	10 (11.6)	14 (7.7)	
$25,000–$34,999	84 (13.8)	24 (15.3)	21 (11.2)	7 (8.1)	32 (17.7)	
$35,000–$49,999	117 (19.2)	29 (18.5)	37 (19.8)	13 (15.1)	38 (21.0)	
$50,000–$74,999	126 (20.6)	28 (17.8)	37 (19.8)	24 (27.9)	37 (20.4)	
More than $75,000	161 (26.4)	46 (29.3)	44 (23.5)	22 (25.6)	49 (27.1)	
Employed						
Yes	375 (61.4)	97 (61.8)	103 (55.1)	49 (57.0)	126 (69.6)	0.0005*
No	122 (20.0)	34 (21.7)	45 (24.1)	9 (10.5)	34 (18.8)	
No, retired	114 (18.7)	26 (16.6)	39 (20.9)	28 (32.6)	21 (11.6)	
Health insurance status						
Insured	525 (85.9)	121 (77.1)	174 (93.1)	76 (88.4)	154 (85.1)	0.0003*
Uninsured	86 (14.1)	36 (22.9)	13 (7.0)	10 (11.6)	27 (14.9)	
Has regular medical care provider						
No	103 (16.9)	33 (21.0)	20 (10.7)	16 (18.6)	34 (18.8)	0.0533
Yes	508 (83.1)	124 (79.0)	167 (89.3)	70 (81.4)	147 (81.2)	
Has seen provider, P12M						
No	92 (15.1)	26 (16.6)	21 (11.2)	11 (12.8)	34 (18.8)	0.1926
Yes	519 (84.9)	131 (83.4)	166 (88.8)	75 (87.2)	147 (81.2)	
Current tobacco smoker						
No	432 (70.7)	108 (68.8)	119 (63.6)	61 (70.9)	144 (79.6)	0.0087*
Yes	179 (29.3)	49 (31.2)	68 (36.4)	25 (29.1)	37 (20.4)	
Family history of cancer						
No	346 (56.6)	84 (53.5)	102 (56.6)	50 (58.1)	110 (60.8)	0.4660
Yes	227 (37.2)	59 (37.6)	77 (37.2)	31 (36.1)	60 (33.2)	
Not sure	38 (6.22)	14 (8.9)	8 (6.2)	5 (5.81)	11 (6.1)	
Family history of colorectal cancer						
No	487 (79.7)	124 (79.0)	141 (75.4)	71 (82.6)	151 (83.4)	0.2029
Yes	53 (8.7)	11 (7.0)	20 (15.2)	10 (11.6)	12 (6.6)	
Not sure	71 (11.6)	22 (14.0)	26 (13.9)	5 (5.8)	18 (9.9)	
Colorectal cancer screening participation						0.5415
Yes	383 (62.7)	92 (58.6)	123 (65.8)	56 (65.12)	112 (61.9)	0.5970
Stool‐based test only	69 (11.3)	17 (10.8)	18 (9.6)	8 (9.3)	26 (14.4)	
Exam‐based test only	135 (22.1)	35 (22.3)	45 (24.1)	17 (19.8)	38 (21.0)	
Both tests	179 (29.3)	40 (25.5)	60 (32.1)	31 (36.1)	48 (26.5)	
No (neither test)	228 (37.3)	65 (41.4)	64 (34.2)	30 (34.9)	69 (38.1)	

*Note*: Frequencies and column percentages are provided. Hispanic/Latinx subgroups included: (1) Mexican (Mexican, Mexican American, Chicano); (2) Puerto Rican; (3) Cuban; and (4) Other Hispanic/Latino (i.e., other than Mexican, Mexican American, Chicano, Puerto Rican, or Cuban). Participation *p*‐value is calculated based on yes versus no for that specific screening test question.

Abbreviations: HS, High School; P12M, Past 12 Months.

* Indicates statistically significant <0.05.

Significant differences were observed by Hispanic/Latino subgroup for survey language (*p* < 0.0001), age group (*p* < 0.0001), state of residence (*p* < 0.0001), sexual orientation (*p* = 0.0044), educational attainment (*p* = 0.0001), employment (*p* = 0.0005), health insurance status (*p* = 0.0003), and tobacco smoking status (*p* = 0.0087). Specifically, participants who self‐identified as Puerto Rican were most likely to complete the survey in English, followed by those self‐identifying as Mexican. Participants who self‐identified as Mexican primarily resided in Texas; Puerto Rican, in New York; and Cuban and other Hispanic/Latino, primarily in Florida. Participants self‐identifying as Cuban were most likely to have a bachelor's degree or higher, followed by those identifying as other Hispanic/Latino. There were no significant differences in household income by subgroup.

As shown in Table 2, participants who had ever completed an exam‐based and/or stool‐based test were more likely to complete the survey in English (*p* = 0.0018), to be aged 60–75 years (*p* < 0.0001), to be retired (*p* < 0.0001), to have health insurance (*p* = 0.0008), to have a regular medical care provider (*p* < 0.0001), and to report seeing their medical care provider in the past 12 months (*p* < 0.0001).

**TABLE 2:** Sociodemographic characteristics by colorectal cancer screening participation.

	Participation history		*p* Value
	No (*n* = 228 [37.3%])	Yes (*n* = 383 [62.7%])	
Survey language			
English	156 (68.4)	305 (79.6)	0.0018*
Spanish	72 (31.6)	78 (20.4)	
Age group (year)			
45–59	185 (81.1)	203 (53.0)	<0.0001*
60–75	43 (18.9)	180 (47.0)	
Residence: State			
New York	64 (28.1)	113 (29.5)	0.0829
Texas	77 (33.8)	98 (25.6)	
Florida	87 (38.2)	172 (44.9)	
Subgroup			
Mexican	65 (28.5)	92 (24.0)	0.5415
Puerto Rican	64 (28.1)	123 (32.1)	
Cuban	30 (13.2)	56 (14.6)	
Other Hispanic	69 (30.3)	112 (29.2)	
Marital status			
Married	113 (49.6)	212 (55.4)	0.0516
Divorced, widowed, or separated	76 (33.3)	93 (24.3)	
Never married	39 (17.1)	78 (20.4)	
Sexual orientation: sexual identity			
Straight/heterosexual	209 (91.7)	345 (90.3)	0.5757
Nonheterosexual identity	19 (8.3)	37 (9.7)	
Educational attainment			
HS or below	69 (30.3)	88 (23.0)	0.1340
Some college	77 (33.8)	146 (38.1)	
Bachelor's degree or higher	82 (36.0)	149 (38.9)	
Household income			
Less than $15,000	24 (10.5)	30 (7.8)	0.5556
$15,000–$24,999	25 (11.0)	44 (11.5)	
$25,000–$34,999	34 (14.9)	50 (13.1)	
$35,000–$49,999	40 (17.5)	77 (20.1)	
$50,000–$74,999	49 (21.5)	77 (20.1)	
More than $75,000	56 (24.6)	105 (27.4)	
Employed			
Yes	161 (70.6)	214 (55.9)	<0.0001*
No	50 (21.9)	72 (18.8)	
No, retired	17 (7.5)	97 (25.3)	
Insurance status			
Insured	182 (79.8)	343 (89.6)	0.0008*
Uninsured	46 (20.2)	40 (10.4)	
Has regular provider			
No	64 (28.1)	39 (10.2)	<0.0001*
Yes	164 (71.9)	344 (89.8)	
Has seen provider, P12M			
No	53 (23.3)	39 (10.2)	<0.0001*
Yes	175 (76.8)	344 (89.8)	
Current smoker			
No	154 (67.5)	278 (72.6)	0.1855
Yes	74 (32.5)	105 (27.4)	
Family history of cancer			
No	124 (54.4)	222 (58.0)	0.5177
Yes	87 (38.2)	140 (36.6)	
Not sure	17 (7.5)	21 (5.5)	
Family history of colorectal cancer			
No	179 (78.5)	308 (80.4)	0.2847
Yes	17 (7.5)	36 (9.4)	
Not sure	32 (14.0)	39 (10.2)	

Abbreviations: HS, High School; P12M, Past 12 Months.

* Indicates statistically significant <0.05.

### 3.2 Masculinity barriers to medical care

Table S1 shows the mean overall MBMC score and mean score for each subscale by subgroup; Table S2, the prevalence of responses to each item on the MBMC scale overall and by subgroup. Most participants (62.5%) reported that the statement “As a provider, I assure the needs of my family are met” was “Completely true” (*Provider Role* subscale). Conversely, most participants reported that the following statements were “Not at all” true: “A problem with my health will go away on its own” (*Health Problem Minimization* subscale, 63.0%); “Having medical exams below the waist would disrespect my sexuality” (*Fear of Being Perceived as Gay* subscale, 82.5%); and “Men of my race are treated like guinea pigs by medical professionals” (*Medical Mistrust* subscale, 65.8%).

We observed differences by Hispanic/Latino subgroup for item b (“As a provider, I take risks for my family even if I may get hurt or put myself in danger”) on the *Provider Role* subscale (*p* = 0.018); item c (“Medical professionals touching me below the waist is fine when it relates to my health”) on the *Fear of Being Perceived as Gay* subscale (*p* = 0.0038); and items c (“Men of my race trust medical professionals”) and d (“Men of my race rarely receive quality medical care”) on the *Medical Mistrust* subscale (*p* = 0.002 and *p* = 0.0002, respectively). For example, men who self‐identified as of other Hispanic/Latino ethnicity were more likely to report taking risks with their own health if it benefited their family (70.2% [Completely true: 40.3%, Mostly true: 29.8%]), compared with those self‐identifying as Mexican (52.2% [Completely true: 36.3%, Mostly true: 15.9%]); Puerto Rican (54.5% [Completely true: 37.4%, Mostly true: 17.1%]); or Cuban (51.2% [Completely true: 32.6%, Mostly true: 18.6%]).

Men self‐identifying as Cuban were least likely to report that health problems below the waist were embarrassing (*Fear of Being Perceived as Gay* subscale, 12.8% [Completely true: 3.5%, Mostly true: 9.3%]), compared with those self‐identifying as Mexican (19.1% [Completely true: 8.9%, Mostly true: 10.2%]); Puerto Rican (21.4% [Completely true: 8.0%, Mostly true: 13.4%]); or other (13.8% [Completely true: 5.5%, Mostly true: 8.3%]). Men self‐identifying as Cuban were least likely to report receiving quality medical care (*Medical Mistrust* subscale, 11.6% [Completely true: 5.8%, Mostly true: 5.8%]), compared with those self‐identifying as Mexican (23.6% [Completely true: 8.3%, Mostly true: 15.3%]); Puerto Rican (27.8% [Completely true: 11.2%, Mostly true: 16.6%]); or other (24.9% [Completely true: 13.81%, Mostly true: 11.05%]). Conversely, men self‐identifying as Cuban were most likely to report trust in healthcare professionals (*Medical Mistrust* subscale, 61.6% [Completely true: 32.6%, Mostly true: 29.1%]), followed by men of other Hispanic/Latino ethnicity (60.8% [Completely true: 27.1%, Mostly true: 33.7%]).

#### 3.2.1 Multivariable logistic regression model results

After adjusting for Hispanic/Latino subgroup, marital status, survey language, age, and health insurance status, masculinity beliefs marginally reduced the odds of CRC screening participation (Table 3). Specifically, among men of other Hispanic/Latino ethnicity, a 1 unit increase on the *Health‐Related Self‐Reliance* subscale was associated with increased odds of screening participation (aOR: 1.06, 95% CI: 0.95, 1.19), while a 1 unit increase on the *Health‐Related Self‐Reliance* subscale was associated with a decreased odds of screening participation for men self‐identifying as Mexican (aOR: 0.90, 95% CI: 0.79, 1.04), Puerto Rican (aOR: 0.89, 95% CI: 0.78, 1.01), or Cuban (aOR: 0.90, 95% CI: 0.75, 1.09). Likewise, among men self‐identifying as Puerto Rican, a 1 unit increase on the *Restrictive Emotionality* subscale was associated with decreased odds of CRC screening participation (aOR: 0.81, 95% CI: 0.71, 0.93). Conversely, although not statistically significant, the inverse relationship is observed for men self‐identifying as Cuban (aOR: 1.09, 95% CI: 0.88, 1.36). A similar pattern was observed for the *Fear of Being Perceived as Gay* Subscale.

**TABLE 3:** Associations of masculinity barriers to medical care (MBMC) and participation in colorectal cancer screening, by Hispanic/Latino subgroup.

	Overall^a^	Hispanic/Latino Subgroup^b^			
		Mexican	Puerto Rican	Cuban	Other Hispanic
	aOR (95% CI)	aOR (95% CI)	aOR (95% CI)	aOR (95% CI)	aOR (95% CI)
Overall MBMC Scale Score	**0.98 (0.96, 1.00)**	1.00 (0.96, 1.04)	**0.94 (0.90, 0.98**)	0.93 (0.87, 1.00)	0.98 (0.95, 1.02)
Provider Role Subscale	0.98 (0.94, 1.03)	0.95 (0.86, 1.05)	1.00 (0.92, 1.09)	0.90 (0.77, 1.06)	1.00 (0.92, 1.09)
Health‐Related Self‐Reliance Subscale	0.97 (0.91, 1.03)	0.90 (0.79, 1.04)	0.89 (0.78, 1.01)	0.90 (0.75, 1.09)	1.06 (0.95, 1.19)
Health Problem Minimization Subscale	0.97 (0.91, 1.02)	1.06 (0.95, 1.18)	0.92 (0.82, 1.03)	0.88 (0.74, 1.05)	0.92 (0.82, 1.03)
Restrictive Emotionality Subscale	0.95 (0.89, 1.02)	1.01 (0.88, 1.17)	**0.81 (0.71, 0.93)**	1.09 (0.88, 1.36)	1.01 (0.88, 1.15)
Fear of Being Perceived as Gay Subscale	0.97 (0.91, 1.03)	1.08 (0.96, 1.21)	**0.87 (0.76, 0.98)**	0.91 (0.75, 1.10)	0.92 (0.82, 1.03)
Medical Mistrust Subscale	0.97 (0.92, 1.03)	1.05 (0.92, 1.18)	0.95 (0.85, 1.05)	0.88 (0.73, 1.05)	0.98 (0.87, 1.09)

*Note*: Bold indicates statistically significant <0.05.

^**a**^ Adjusted for Hispanic/Latino subgroup, marital status, survey language, age group, and health insurance status.

^**b**^ Adjusted for marital status, survey language, age group, and health insurance status.

## 4 DISCUSSION

Over the past decade, progress in the analysis of CRC screening uptake among Hispanic/Latinx adults, particularly men, has been limited, highlighting an important opportunity to potentially reduce inequities in CRC mortality. To our knowledge, this cross‐sectional study is one of the first to examine variations in CRC early‐detection screening behaviors and links between masculinity beliefs and CRC screening participation among ethnic subgroups of Hispanic/Latino men. Our survey‐based study of 611 men from ethnically and geographically diverse Hispanic/Latino subgroups across three states identified three key findings that help to fill gaps in the literature.

Over half of our sample (62.7%) reported CRC screening participation which is line with national studies examining up‐to‐date status among Hispanic/Latino adults (e.g., 2020 BRFSS estimated 64%).^42^ Although we observed no statistically significant differences in screening behaviors between men who self‐identified as Mexican, Puerto Rican, Cuban, or other Hispanic/Latino ethnicity, our findings are consistent with literature demonstrating that masculinity barriers may contribute to low CRC screening uptake among Hispanic/Latino men.^43^, ^44^ Studies have found that colonoscopy uptake is lower and that of stool‐based testing is higher among Hispanic/Latino men compared with NHL men,^45^, ^46^ and that Hispanic/Latino men are more likely to complete a stool‐based test than a colonoscopy, as the stool‐based test is less invasive and may be perceived as not violating masculine self‐perceptions.^3^, ^8^, ^47^ Viramontes and colleagues found that stool‐based exams were more common among Spanish‐speaking Hispanic/Latino men than among those who primarily speak English.^16^ Warranting further investigation, it has been suggested that some medical providers may not recommend CRC early‐detection screening to Spanish‐speaking males because they believe these men lack health insurance that would cover the screening test.^48^ Future research is warranted that further investigates CRC early‐detection screening behaviors among Spanish versus English speakers as well as among men from the 10 largest Hispanic‐origin groups—Colombians, Cubans, Dominicans, Ecuadorians, Guatemalans, Hondurans, Mexicans, Peruvians, Puerto Ricans, and Salvadorans—that make up 92% of the US Hispanic/Latinx population.^3^, ^16^

We found that for men in our study, masculinity barriers may hinder CRC screening participation. Specifically, a strong relationship was observed among Puerto Rican men holding back emotions (*Restrictive Emotionality* subscale) and lower odds of screening participation. On the contrary, among men of other Hispanic/Latino ethnicity, those who felt more confident in managing their health (*Health‐Related Self‐Reliance* subscale) were more likely to participate in CRC screening. Our findings are consistent with prior research in other racial/ethnic groups, including Black and American Indian/Alaskan Native men, showing that being strong and having negative attitudes toward medical professionals and exams were associated with lower odds of CRC screening participation.^22^, ^38^, ^39^, ^49^ Across multiple racial and ethnic backgrounds, men who strongly identify with masculine ideals or patriarchal values demonstrate reluctance to engage in preventive health behaviors.^22^, ^32^, ^38^, ^49^ These health behaviors are rooted in fears of being perceived as weak or vulnerable by their families, peers, or broader communities.^50^, ^51^, ^52^

Prior qualitative work has explored Hispanic/Latino men's ideals related to masculinity and attributes that demonstrate their character and cultural values.^50^ In general, among these men, the concept of manhood is closely intertwined with the importance of reflecting salient cultural values of accountability, specifically to their children and elders, and is also closely related to devotion to family and to the ability to provide for one's family financially. *Machismo*, a social construct closely related to masculinity that has been explored among Mexican and Mexican American men, is defined as adherence to a code of ethics that guides behavior while emphasizing that men are heterogeneous and is highly dependent on an individual's context.^53^ Limited prior qualitative research has demonstrated that machismo is a barrier to CRC screening due to embarrassment or unwillingness to undergo a rectal examination.^54^ Future studies should further explore the magnitude of the association of machismo with the presently examined masculinity barriers among our study population.

Stratification by Hispanic/Latino subgroup revealed differences on some measures on the MBMC scale. First, while Cuban men were most likely to report trust in healthcare professionals, they were least likely to report receiving quality medical care. Compared with many high‐income countries, Cuba has a reputation for offering Cubans longer life expectancies and better health‐care access due to its well‐performing health system.^55^ Existing literature suggests that Cuban men may hold comparable expectations for the US health system, but may not be receiving care of the desired quality. Complex interactions between payers, providers, and male Cuban patients may also contribute to these men's receipt of substandard medical care.^56^ Previous studies have laid the foundation for the steps necessary to improve care quality among Hispanic/Latino men of Cuban ethnicity, which includes health systems enhancing patient satisfaction with care and service, restructuring delivery systems, providing financial incentives to ensure the quality of public health and primary care services (e.g., quality add‐on payments, provider performance bonuses, or penalties), and expanding the supply of culturally competent providers who are rewarded for spending time with patients.^57^, ^58^

Another masculinity‐focused finding was that men of other Hispanic/Latino ethnicity were more likely to report taking risks with their own health if it benefited their family and that health problems below the waist were embarrassing. Similarly, Getrich et al. reported that Mexican men saw colonoscopies as embarrassing, and that the procedure may trigger alpha male characteristics of dominance and strength stemming from machismo culture, as the study participants viewed exams involving the rectum and anus as forbidden.^44^ Future studies should focus on determining how best to encourage providers of CRC screening to address machismo in a culturally sensitive manner by improving patient‐provider communication and patient education targeted to Hispanic/Latino men. Additionally, while the MBMC's validity specifically in Hispanic/Latino populations should be further examined, our findings should inform tailored interventions, by subgroups and by masculinity subscales, aimed at increasing CRC screening participation in this population.

## 5 LIMITATIONS

This study advances efforts to address knowledge gaps regarding CRC early‐detection screening among Hispanic/Latino men. Nevertheless, our findings should be interpreted in the context of several limitations. First, we relied on self‐reported survey data that may be more susceptible to misclassification (information bias) than other data sources. Nonetheless, self‐reported screening participation is generally considered valid and reliable, and would likely bias our results toward the null.^59^, ^60^ Additionally, our survey question did not explicitly use the term “screening,” thus we were unable to distinguish screening from diagnostic tests. However, we believe this likely did not substantially affect our results as this phrasing aligns with national surveys widely used in behavioral and chronic disease surveillance (e.g., Behavioral Risk Factor Surveillance System and National Health Interview Survey).

Second, while the MBMC scale has been psychometrically assessed among African‐American, White, and Indigenous men, its direct validation within our study subgroup, Hispanic/Latino men, has not been established.^38^ Although efforts were made to ensure the relevance and appropriateness of the tool for our population, its application in this specific subgroup may have introduced inherent limitations that warrant further investigation. Future studies should prioritize additional validation of the tool within our study population to strengthen the robustness of findings and inform future interventions.

Third, our measurement of language (a common acculturation proxy) aimed to balance participant burden and financial constraints but may not fully capture acculturation changes within individual social networks. Given the established link between acculturation and health behaviors among Hispanic/Latinx people, unmeasured confounding could be present.^61^, ^62^, ^63^, ^64^ Consideration of additional factors like language proficiency, foreign‐born status, length of US residence, generational status, or residency in Hispanic/Latinx enclaves might be crucial.

Fourth, our sample size limited the extent of analysis that could be conducted and the small sample sizes of other Hispanic/Latinx ethnic groups may have restricted our ability to distinguish between‐group variations. Nonetheless, we achieved our novel exploration of differences among major Hispanic/Latino ethnic subgroups (Mexican, Puerto Rican, and Cuban) that are understudied in the CRC literature and are often not disaggregated.

Fifth, because our target population resided in only three US states, our findings may not be generalizable to Hispanic/Latino men residing in other states (e.g., California and Arizona).^36^ Moreover, we cannot account for the possibility that political controversies occurring around the time of data collection in the states in which our study participants resided (e.g., concerns in Texas related to the border with Mexico, recurring issues in Florida related to the state's Hispanic/Latinx population, the transportation of undocumented immigrants from Florida and Texas to New York) may have influenced healthcare engagement and decisions to complete CRC screening. Nonetheless, our findings offer a basis for future hypothesis generation, as the states we recruited from are among the five US states with the highest Hispanic/Latinx populations.^65^

Lastly, since we recruited our study participants via a consumer panel, our sample was limited to individuals with internet access (90% of Americans in 2022^66^) and was self‐selected to those willing to participate in market research and who consented to the study. Thus, our sample may lack external validity, be subject to type 2 selection bias, and for these reasons may be unrepresentative of the target population.^67^ However, several prior studies have demonstrated that consumer panels are a reliable method of recruitment for collecting survey data that are demographically representative based on criteria such as gender, race and ethnicity, age, socioeconomic status, and for surveying populations that researchers describe as “hard‐to‐reach.”^33^, ^34^, ^68^

## 6 CONCLUSIONS

We found low uptake of CRC screening among Hispanic/Latino men, with over 40% of eligible men reporting no history of screening participation. We did not observe differences in CRC screening participation by ethnic subgroup, suggesting that barriers to screening may be similar among Hispanic/Latino men generally. Men with Spanish as their preferred language and markers of poor access to care (e.g., being uninsured) should be prioritized for future intervention. Culturally appropriate approaches that incorporate concepts of machismo may be effective in addressing barriers to CRC screening among Hispanic/Latino men. Gaining a deeper comprehension of how masculinity beliefs influence Hispanic/Latino subgroups' decision‐making regarding CRC screening and preventive healthcare is vital. Intervention strategies that specifically target these beliefs could significantly decrease CRC‐associated mortality rates among Hispanic/Latino men.

## AUTHOR CONTRIBUTIONS

**Ami Elizabeth Sedani:** Data curation (supporting); formal analysis (lead); methodology (lead); supervision (lead); validation (lead); visualization (lead); writing – original draft (lead). **Jessica Yasmine Islam:** Conceptualization (equal); data curation (lead); funding acquisition (equal); investigation (equal); methodology (supporting); writing – original draft (supporting). **Derek M. Griffith:** Writing – original draft (supporting). **Kelly Krupa Rifelj:** Writing – original draft (supporting). **Cordero McCall:** Writing – original draft (supporting). **Omar García‐Rodríguez:** Writing – original draft (supporting). **Marlene Camacho‐Rivera:** Conceptualization (equal); funding acquisition (equal). **Charles Ray Rogers:** Conceptualization (equal); funding acquisition (equal); methodology (supporting); project administration (lead); supervision (supporting); writing – original draft (supporting).

## CONFLICT OF INTEREST STATEMENT

Although unrelated to this study, Dr. Charles R. Rogers offers scientific input to research studies through an investigator services agreement with Exact Sciences. No other authors have any conflicts of interest to declare.

## ETHICS STATEMENT

This research was conducted ethically in accordance with the World Medical Association Declaration of Helsinki. This study was approved by the Institutional Review Board (IRB) at the University of Utah (IRB #00113679).

## Supporting information


Appendix S1.

## Data Availability

Data supporting the study's findings are available upon reasonable request from the second author [JYI].
