# Supplementary material for: Sociocultural and masculinity influences on colorectal cancer screening participation among Hispanic/Latino men in Florida, New York, and Texas
Source: Cancer Med. 2024 Sep 20;13(18):e70159. doi: 10.1002/cam4.70159 (PMC11413917; doi:10.1002/cam4.70159)
Supplement: Supplementary file 1 — Appendix S1. [file CAM4-13-e70159-s001.docx]

**Supplementary Material**

**Sociocultural and Masculinity Influences on Colorectal Cancer Screening Uptake among Hispanic/Latino Men in Florida, New York, and Texas**

**Figure S1**. Masculinity barriers to medical care (MBMC) scale, subscales, and items.

**
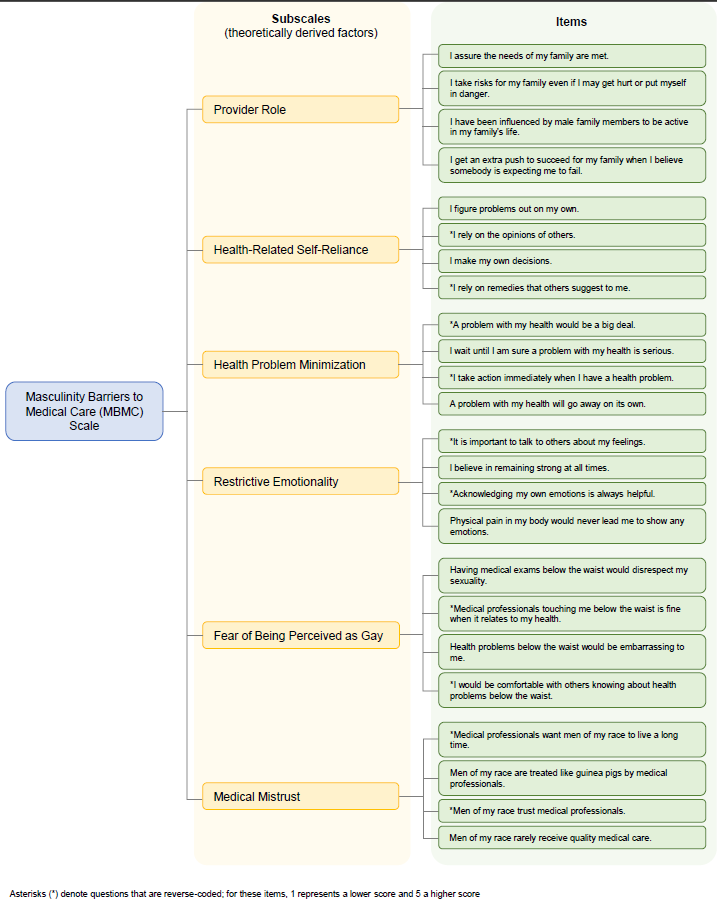
**

**Table S1.** Masculinity barriers to medical care (MBMC) scale measures among Hispanic/Latino men aged 45+ years residing in FL, NY, TX (February – March 2022).

|  | **Total**  (n=611) | | **Mexican**  (n=157 [25.7%]) | | **Puerto Rican**  (n=187 [30.6%]) | | **Cuban**  (n=86 [14.1%]) | | **Other**  **Hispanic/Latino**  (n=181 [29.6%]) | | **P-Value** |
| --- | --- | --- | --- | --- | --- | --- | --- | --- | --- | --- | --- |
| **MBMC Subscales and Items** | No. | Col % | No. | Col % | No. | Col % | No. | Col % | No. | Col % |  |
| **Subscale 1: Provider Role** |  |  |  |  |  |  |  |  |  |  | 0.5933 |
| **1a. As a provider, I assure the needs of my family are met.** |  |  |  |  |  |  |  |  |  |  | 0.5901 |
| Not at all | 15 | 2.5 | 2 | 1.3 | 4 | 2.1 | 3 | 3.5 | 6 | 3.3 |  |
| Slightly True | 23 | 3.8 | 9 | 5.7 | 6 | 3.2 | 2 | 2.3 | 6 | 3.3 |  |
| Moderately True | 50 | 8.2 | 11 | 7.0 | 19 | 10.2 | 7 | 8.1 | 13 | 7.2 |  |
| Mostly True | 141 | 23.1 | 39 | 24.8 | 35 | 18.7 | 26 | 30.2 | 41 | 22.7 |  |
| Completely True | 382 | 62.5 | 96 | 61.2 | 123 | 65.8 | 48 | 55.8 | 115 | 63.5 |  |
| **1b. As a provider, I take risks for my family even if I may get hurt or put myself in danger.** |  |  |  |  |  |  |  |  |  |  | 0.0180* |
| Not at all | 96 | 15.7 | 25 | 15.9 | 34 | 18.2 | 14 | 16.3 | 23 | 12.7 |  |
| Slightly True | 62 | 10.2 | 23 | 14.7 | 18 | 9.6 | 9 | 10.5 | 12 | 6.6 |  |
| Moderately True | 98 | 16.0 | 27 | 17.2 | 33 | 17.7 | 19 | 22.1 | 19 | 10.5 |  |
| Mostly True | 127 | 20.8 | 25 | 15.9 | 32 | 17.1 | 16 | 18.6 | 54 | 29.8 |  |
| Completely True | 228 | 37.3 | 57 | 36.3 | 70 | 37.4 | 28 | 32.6 | 73 | 40.3 |  |
| **1c. As a provider, I have been influenced by male family members to be active in my family's life.** |  |  |  |  |  |  |  |  |  |  | 0.4800 |
| Not at all | 126 | 20.6 | 29 | 18.5 | 35 | 18.7 | 22 | 25.6 | 40 | 22.1 |  |
| Slightly True | 58 | 9.5 | 20 | 12.7 | 16 | 8.6 | 6 | 7.0 | 16 | 8.8 |  |
| Moderately True | 114 | 18.7 | 21 | 13.4 | 41 | 21.9 | 19 | 22.1 | 33 | 18.2 |  |
| Mostly True | 146 | 23.9 | 42 | 26.8 | 42 | 22.5 | 15 | 17.4 | 47 | 26.0 |  |
| Completely True | 167 | 27.3 | 45 | 28.7 | 53 | 28.3 | 24 | 27.9 | 45 | 24.9 |  |
| **1d. As a provider, I get an extra push to succeed for my family when I believe somebody is expecting me to fail.** |  |  |  |  |  |  |  |  |  |  | 0.8009 |
| Not at all | 106 | 17.4 | 25 | 15.9 | 30 | 16.0 | 17 | 19.8 | 34 | 18.8 |  |
| Slightly True | 52 | 8.5 | 19 | 12.1 | 14 | 7.5 | 5 | 5.8 | 14 | 7.7 |  |
| Moderately True | 94 | 15.4 | 25 | 15.9 | 32 | 17.1 | 14 | 16.3 | 23 | 12.7 |  |
| Mostly True | 138 | 22.6 | 37 | 23.6 | 39 | 20.9 | 22 | 25.6 | 40 | 22.1 |  |
| Completely True | 221 | 36.2 | 51 | 32.5 | 72 | 38.5 | 28 | 32.6 | 70 | 38.7 |  |
| **Subscale 2: Health Related Self Reliance** |  |  |  |  |  |  |  |  |  |  | 0.7065 |
| **2a. When it comes to my health, I figure problems out on my own.** |  |  |  |  |  |  |  |  |  |  | 0.9823 |
| Not at all | 132 | 21.6 | 35 | 22.3 | 37 | 19.8 | 20 | 23.3 | 40 | 22.1 |  |
| Slightly True | 103 | 16.9 | 27 | 17.2 | 34 | 18.2 | 15 | 17.4 | 27 | 14.9 |  |
| Moderately True | 169 | 27.7 | 45 | 28.7 | 50 | 26.7 | 25 | 29.1 | 49 | 27.1 |  |
| Mostly True | 127 | 20.8 | 34 | 21.7 | 41 | 21.9 | 16 | 18.6 | 36 | 19.9 |  |
| Completely True | 80 | 13.1 | 16 | 10.2 | 25 | 13.4 | 10 | 11.6 | 29 | 16.0 |  |
| **2b. When it comes to my health, I rely on the opinions of others.** ^†^ |  |  |  |  |  |  |  |  |  |  | 0.6708 |
| Completely True | 41 | 6.7 | 12 | 7.6 | 12 | 6.4 | 7 | 8.1 | 10 | 5.5 |  |
| Mostly True | 84 | 13.8 | 22 | 14 | 21 | 11.2 | 15 | 17.4 | 26 | 14.4 |  |
| Moderately True | 128 | 21.0 | 32 | 20.4 | 37 | 19.8 | 21 | 24.4 | 38 | 21.0 |  |
| Slightly True | 161 | 26.4 | 41 | 26.1 | 61 | 32.6 | 16 | 18.6 | 43 | 23.8 |  |
| Not at all | 197 | 32.2 | 50 | 31.9 | 56 | 30 | 27 | 31.4 | 64 | 35.4 |  |
| **2c. When it comes to my health, I make my own decisions.** |  |  |  |  |  |  |  |  |  |  | 0.9028 |
| Not at all | 35 | 5.7 | 11 | 7.0 | 8 | 4.3 | 6 | 7.0 | 10 | 5.5 |  |
| Slightly True | 63 | 10.3 | 17 | 10.8 | 21 | 11.2 | 10 | 11.6 | 15 | 8.3 |  |
| Moderately True | 83 | 13.6 | 21 | 13.4 | 25 | 13.4 | 15 | 17.4 | 22 | 12.2 |  |
| Mostly True | 200 | 32.7 | 50 | 31.9 | 61 | 32.6 | 30 | 34.9 | 59 | 32.6 |  |
| Completely True | 230 | 37.6 | 58 | 36.9 | 72 | 38.5 | 25 | 29.1 | 75 | 41.4 |  |
| **2d. When it comes to my health, I rely on remedies that others suggest to me.**^†^ |  |  |  |  |  |  |  |  |  |  | 0.6055 |
| Completely True | 218 | 35.7 | 46 | 29.3 | 61 | 32.6 | 35 | 40.7 | 76 | 42.0 |  |
| Mostly True | 182 | 29.8 | 48 | 30.6 | 60 | 32.1 | 27 | 31.4 | 47 | 26.0 |  |
| Moderately True | 132 | 21.6 | 40 | 25.5 | 40 | 21.4 | 15 | 17.4 | 37 | 20.4 |  |
| Slightly True | 53 | 8.7 | 16 | 10.2 | 16 | 8.6 | 7 | 8.1 | 14 | 7.7 |  |
| Not at all | 26 | 4.3 | 7 | 4.5 | 10 | 5.4 | 2 | 2.3 | 7 | 3.9 |  |
| **Subscale 3: Health Problem Minimization** |  |  |  |  |  |  |  |  |  |  |  |
| **3a. A problem with my health would be a big deal.** ^†^ |  |  |  |  |  |  |  |  |  |  | 0.899 |
| Completely True | 46 | 7.5 | 13 | 8.3 | 8 | 4.3 | 8 | 9.3 | 17 | 9.4 |  |
| Mostly True | 62 | 10.2 | 16 | 10.2 | 17 | 9.1 | 8 | 9.3 | 21 | 11.6 |  |
| Moderately True | 105 | 17.2 | 27 | 17.2 | 35 | 18.7 | 15 | 17.4 | 28 | 15.5 |  |
| Slightly True | 144 | 23.6 | 37 | 23.6 | 44 | 23.5 | 18 | 20.9 | 45 | 24.9 |  |
| Not at all | 254 | 41.6 | 64 | 40.8 | 83 | 44.4 | 37 | 43.0 | 70 | 38.7 |  |
| **3b. I wait until I am sure a problem with my health is serious.** |  |  |  |  |  |  |  |  |  |  | 0.4352 |
| Not at all | 204 | 33.4 | 51 | 32.5 | 58 | 31.0 | 36 | 41.9 | 59 | 32.6 |  |
| Slightly True | 103 | 16.9 | 25 | 15.9 | 28 | 15.0 | 15 | 17.4 | 35 | 19.34 |  |
| Moderately True | 140 | 22.9 | 39 | 24.8 | 46 | 24.6 | 17 | 19.8 | 38 | 20.99 |  |
| Mostly True | 110 | 18.0 | 25 | 15.9 | 43 | 23.0 | 13 | 15.1 | 29 | 16.02 |  |
| Completely True | 54 | 8.8 | 17 | 10.8 | 12 | 6.4 | 5 | 5.8 | 20 | 11.05 |  |
| **3c. I take action immediately when I have a health problem.** ^†^ |  |  |  |  |  |  |  |  |  |  | 0.1945 |
| Completely True | 31 | 5.1 | 10 | 6.4 | 10 | 5.4 | 4 | 4.7 | 7 | 3.87 |  |
| Mostly True | 105 | 17.2 | 35 | 22.3 | 38 | 20.3 | 13 | 15.1 | 19 | 10.5 |  |
| Moderately True | 101 | 16.5 | 28 | 17.8 | 31 | 16.6 | 11 | 12.8 | 31 | 17.13 |  |
| Slightly True | 151 | 24.7 | 38 | 24.2 | 41 | 21.9 | 25 | 29.1 | 47 | 25.97 |  |
| Not at all | 223 | 36.5 | 46 | 29.3 | 67 | 35.8 | 33 | 38.4 | 77 | 42.54 |  |
| **3d. A problem with my health will go away on its own.** |  |  |  |  |  |  |  |  |  |  | 0.3189 |
| Not at all | 385 | 63.0 | 91 | 58.0 | 115 | 61.5 | 61 | 70.9 | 118 | 65.19 |  |
| Slightly True | 95 | 15.6 | 28 | 17.8 | 31 | 16.6 | 12 | 14.0 | 24 | 13.26 |  |
| Moderately True | 82 | 13.4 | 20 | 12.7 | 30 | 16.0 | 8 | 9.3 | 24 | 13.26 |  |
| Mostly True | 30 | 4.9 | 11 | 7.0 | 4 | 2.1 | 5 | 5.8 | 10 | 5.52 |  |
| Completely True | 19 | 3.1 | 7 | 4.5 | 7 | 3.7 | 0 | 0.0 | 5 | 2.76 |  |
| **Subscale 4: Restrictive Emotionality** |  |  |  |  |  |  |  |  |  |  |  |
| **4a. It is important to talk to others about my  feelings.** ^†^ |  |  |  |  |  |  |  |  |  |  | 0.5314 |
| Completely True | 64 | 10.5 | 20 | 12.7 | 19 | 10.2 | 8 | 9.3 | 17 | 9.39 |  |
| Mostly True | 126 | 20.6 | 31 | 19.8 | 39 | 20.9 | 22 | 25.6 | 34 | 18.78 |  |
| Moderately True | 172 | 28.2 | 49 | 31.2 | 49 | 26.2 | 25 | 29.1 | 49 | 27.07 |  |
| Slightly True | 152 | 24.9 | 39 | 24.8 | 44 | 23.5 | 15 | 17.4 | 54 | 29.83 |  |
| Not at all | 97 | 15.9 | 18 | 11.5 | 36 | 19.3 | 16 | 18.6 | 27 | 14.92 |  |
| **4b. I believe in remaining strong at all times.** |  |  |  |  |  |  |  |  |  |  | 0.5642 |
| Not at all | 12 | 2.0 | 4 | 2.6 | 3 | 1.6 | 3 | 3.5 | 2 | 1.1 |  |
| Slightly True | 53 | 8.7 | 16 | 10.2 | 16 | 8.6 | 6 | 7.0 | 15 | 8.29 |  |
| Moderately True | 130 | 21.3 | 30 | 19.1 | 35 | 18.7 | 23 | 26.7 | 42 | 23.2 |  |
| Mostly True | 174 | 28.5 | 46 | 29.3 | 46 | 24.6 | 26 | 30.2 | 56 | 30.94 |  |
| Completely True | 242 | 39.6 | 61 | 38.9 | 87 | 46.5 | 28 | 32.6 | 66 | 36.46 |  |
| **4c. Acknowledging my own emotions is always helpful.** ^†^ |  |  |  |  |  |  |  |  |  |  | 0.3420 |
| Completely True | 19 | 3.1 | 5 | 3.2 | 9 | 4.8 | 2 | 2.3 | 3 | 1.66 |  |
| Mostly True | 51 | 8.4 | 19 | 12.1 | 15 | 8.0 | 3 | 3.5 | 14 | 7.73 |  |
| Moderately True | 136 | 22.3 | 38 | 24.2 | 38 | 20.3 | 22 | 25.6 | 38 | 20.99 |  |
| Slightly True | 184 | 30.1 | 49 | 31.2 | 53 | 28.3 | 29 | 33.7 | 53 | 29.28 |  |
| Not at all | 221 | 36.2 | 46 | 29.3 | 72 | 38.5 | 30 | 34.9 | 73 | 40.33 |  |
| **4d. Physical pain in my body would never lead me to show any emotions.** |  |  |  |  |  |  |  |  |  |  | 0.5956 |
| Not at all | 197 | 32.2 | 55 | 35.0 | 55 | 29.4 | 27 | 31.4 | 60 | 33.15 |  |
| Slightly True | 118 | 19.3 | 29 | 18.5 | 38 | 20.3 | 20 | 23.3 | 31 | 17.13 |  |
| Moderately True | 150 | 24.6 | 43 | 27.4 | 39 | 20.9 | 22 | 25.6 | 46 | 25.41 |  |
| Mostly True | 95 | 15.6 | 21 | 13.4 | 33 | 17.7 | 13 | 15.1 | 28 | 15.47 |  |
| Completely True | 51 | 8.4 | 9 | 5.7 | 22 | 11.8 | 4 | 4.7 | 16 | 8.84 |  |
| **Subscale 5: Fear of Being Perceived as Gay** |  |  |  |  |  |  |  |  |  |  |  |
| **5a. Having medical exams below the waist would disrespect my sexuality.** |  |  |  |  |  |  |  |  |  |  | 0.6556 |
| Not at all | 504 | 82.5 | 131 | 83.4 | 146 | 78.1 | 71 | 82.6 | 156 | 86.19 |  |
| Slightly True | 33 | 5.4 | 6 | 3.8 | 14 | 7.5 | 4 | 4.7 | 9 | 4.97 |  |
| Moderately True | 28 | 4.6 | 9 | 5.7 | 12 | 6.4 | 4 | 4.7 | 3 | 1.66 |  |
| Mostly True | 22 | 3.6 | 4 | 2.6 | 8 | 4.3 | 4 | 4.7 | 6 | 3.31 |  |
| Completely True | 24 | 3.93 | 7 | 4.5 | 7 | 3.7 | 3 | 3.5 | 7 | 3.87 |  |
| **5b. Medical professionals touching me below the waist is fine when it relates to my health.** ^†^ |  |  |  |  |  |  |  |  |  |  | 0.3763 |
| Completely True | 32 | 5.2 | 10 | 6.4 | 7 | 3.7 | 5 | 5.8 | 10 | 5.5 |  |
| Mostly True | 34 | 5.6 | 8 | 5.1 | 10 | 5.4 | 7 | 8.1 | 9 | 5.0 |  |
| Moderately True | 53 | 8.7 | 15 | 9.6 | 25 | 13.4 | 5 | 5.8 | 8 | 4.4 |  |
| Slightly True | 122 | 20.0 | 32 | 20.4 | 35 | 18.7 | 16 | 18.6 | 39 | 21.6 |  |
| Not at all | 370 | 60.6 | 92 | 58.6 | 110 | 58.8 | 53 | 61.6 | 115 | 63.5 |  |
| **5c. Health problems below the waist would be embarrassing to me.** |  |  |  |  |  |  |  |  |  |  | 0.0038* |
| Not at all | 290 | 47.5 | 66 | 42.0 | 69 | 36.9 | 49 | 57.0 | 106 | 58.6 |  |
| Slightly True | 119 | 19.5 | 30 | 19.1 | 42 | 22.5 | 20 | 23.3 | 27 | 14.9 |  |
| Moderately True | 96 | 15.7 | 31 | 19.8 | 36 | 19.3 | 6 | 7.0 | 23 | 12.7 |  |
| Mostly True | 64 | 10.5 | 16 | 10.2 | 25 | 13.4 | 8 | 9.3 | 15 | 8.3 |  |
| Completely True | 42 | 6.87 | 14 | 8.9 | 15 | 8.0 | 3 | 3.5 | 10 | 5.5 |  |
| **5d. I would be comfortable with others knowing about health problems below the waist.** ^†^ |  |  |  |  |  |  |  |  |  |  | 0.1233 |
| Completely True | 195 | 31.9 | 60 | 38.2 | 62 | 33.2 | 26 | 30.2 | 47 | 26.0 |  |
| Mostly True | 111 | 18.2 | 34 | 21.7 | 31 | 16.6 | 15 | 17.4 | 31 | 17.1 |  |
| Moderately True | 128 | 21.0 | 28 | 17.8 | 47 | 25.1 | 17 | 19.8 | 36 | 19.9 |  |
| Slightly True | 90 | 14.7 | 19 | 12.1 | 22 | 11.8 | 17 | 19.8 | 32 | 17.7 |  |
| Not at all | 87 | 14.2 | 16 | 10.2 | 25 | 13.4 | 11 | 12.8 | 35 | 19.3 |  |
| **Subscale 6: Medical Mistrust** |  |  |  |  |  |  |  |  |  |  | 0.4973 |
| **6a. Medical professionals want men of my race to live a long time.** ^†^ |  |  |  |  |  |  |  |  |  |  | 0.5465 |
| Completely True | 85 | 13.9 | 17 | 10.8 | 25 | 13.4 | 15 | 17.4 | 28 | 15.47 |  |
| Mostly True | 50 | 8.2 | 11 | 7.0 | 17 | 9.1 | 7 | 8.1 | 15 | 8.29 |  |
| Moderately True | 148 | 24.2 | 40 | 25.5 | 52 | 27.8 | 20 | 23.3 | 36 | 19.89 |  |
| Slightly True | 144 | 23.6 | 43 | 27.4 | 41 | 21.9 | 13 | 15.1 | 47 | 25.97 |  |
| Not at all | 184 | 30.1 | 46 | 29.3 | 52 | 27.8 | 31 | 36.1 | 55 | 30.39 |  |
| **6b. Men of my race are treated like guinea pigs by medical professionals.** |  |  |  |  |  |  |  |  |  |  | 0.3504 |
| Not at all | 402 | 65.79 | 108 | 68.8 | 111 | 59.4 | 67 | 77.9 | 116 | 64.09 |  |
| Slightly True | 71 | 11.62 | 19 | 12.1 | 25 | 13.4 | 8 | 9.3 | 19 | 10.5 |  |
| Moderately True | 88 | 14.4 | 17 | 10.8 | 33 | 17.7 | 9 | 10.5 | 29 | 16.02 |  |
| Mostly True | 28 | 4.58 | 7 | 4.5 | 11 | 5.9 | 1 | 1.2 | 9 | 4.97 |  |
| Completely True | 22 | 3.6 | 6 | 3.8 | 7 | 3.7 | 1 | 1.2 | 8 | 4.42 |  |
| **6c. Men of my race trust medical professionals.** ^†^ |  |  |  |  |  |  |  |  |  |  | 0.0020* |
| Completely True | 53 | 8.67 | 25 | 15.9 | 14 | 7.5 | 5 | 5.8 | 9 | 4.97 |  |
| Mostly True | 94 | 15.38 | 29 | 18.5 | 35 | 18.7 | 9 | 10.5 | 21 | 11.6 |  |
| Moderately True | 146 | 23.9 | 39 | 24.8 | 47 | 25.1 | 19 | 22.1 | 41 | 22.65 |  |
| Slightly True | 177 | 28.97 | 41 | 26.1 | 50 | 26.7 | 25 | 29.1 | 61 | 33.7 |  |
| Not at all | 141 | 23.08 | 23 | 14.7 | 41 | 21.9 | 28 | 32.6 | 49 | 27.07 |  |
| **6d. Men of my race rarely receive quality medical care.** |  |  |  |  |  |  |  |  |  |  | 0.0002* |
| Not at all | 240 | 39.28 | 60 | 38.2 | 57 | 30.5 | 55 | 64.0 | 68 | 37.57 |  |
| Slightly True | 100 | 16.37 | 27 | 17.2 | 35 | 18.7 | 14 | 16.3 | 24 | 13.26 |  |
| Moderately True | 127 | 20.79 | 33 | 21.0 | 43 | 23.0 | 7 | 8.1 | 44 | 24.31 |  |
| Mostly True | 80 | 13.09 | 24 | 15.3 | 31 | 16.6 | 5 | 5.8 | 20 | 11.05 |  |
| Completely True | 64 | 10.47 | 13 | 8.3 | 21 | 11.2 | 5 | 5.8 | 25 | 13.81 |  |

**Note**:

*Statistically significant <0.05

^†^questions that are reverse-coded; for these items, 1 represents a lower score and 5 a higher score.
Abbreviations: MBMC = Masculinity barriers to medical care; No = Number; Col = Column.

**Table S2.** Masculinity barriers to medical care scale (MBMC) mean scores, by Hispanic/Latino subgroup.

| **MBMC Subscales and Items** | **Total**  (n=611) | | **Mexican**  (n=157) | | **Puerto Rican**  (n=187) | | **Cuban**  (n=86) | | **Other**  **Hispanic/Latino**  (n=181) | |
| --- | --- | --- | --- | --- | --- | --- | --- | --- | --- | --- |
|  | **mean** | **std** | **mean** | **std** | **mean** | **std** | **mean** | **std** | **mean** | **std** |
| **Subscale 1: Provider Role** | 3.7 | 0.9 | 3.6 | 0.9 | 3.7 | 0.9 | 3.6 | 1.0 | 3.7 | 0.9 |
| **1a. As a provider, I assure the needs of my family are met.** | 4.4 | 1.0 | 4.4 | 0.9 | 4.4 | 0.9 | 4.3 | 1.0 | 4.4 | 1.0 |
| **1b. As a provider, I take risks for my family even if I may get hurt or put myself in danger** | 3.5 | 1.5 | 3.4 | 1.5 | 3.5 | 1.5 | 3.4 | 1.5 | 3.8 | 1.4 |
| **1c. As a provider, I have been influenced by male family members to be active in my family's life.** | 3.3 | 1.5 | 3.3 | 1.5 | 3.3 | 1.4 | 3.2 | 1.5 | 3.2 | 1.5 |
| **1d. As a provider, I get an extra push to succeed for my family when I believe somebody is expecting me to fail.** | 3.5 | 1.5 | 3.4 | 1.5 | 3.6 | 1.5 | 3.5 | 1.5 | 3.5 | 1.5 |
| **Subscale 2: Health Related Self Reliance** | 3.6 | 0.7 | 3.5 | 0.7 | 3.6 | 0.7 | 3.5 | 0.8 | 3.6 | 0.7 |
| **2a. When it comes to my health, I figure problems out on my own.** | 2.9 | 1.3 | 2.8 | 1.3 | 2.9 | 1.3 | 2.8 | 1.3 | 2.9 | 1.4 |
| **2b. When it comes to my health, I rely on the opinions of others.** | 3.6 | 1.2 | 3.6 | 1.3 | 3.7 | 1.2 | 3.5 | 1.3 | 3.7 | 1.2 |
| **2c. When it comes to my health, I make my own decisions.** | 3.9 | 1.2 | 3.8 | 1.2 | 3.9 | 1.2 | 3.7 | 1.2 | 4.0 | 1.2 |
| **2d. When it comes to my health, I rely on remedies that others suggest to me.** | 3.8 | 1.1 | 3.7 | 1.1 | 3.8 | 1.2 | 4.0 | 1.1 | 3.9 | 1.1 |
| **Subscale 3: Health Problem Minimization** | 2.2 | 0.8 | 2.3 | 0.8 | 2.2 | 0.7 | 2.0 | 0.8 | 2.1 | 0.7 |
| **3a. A problem with my health would be a big deal.** | 2.2 | 1.3 | 2.2 | 1.3 | 2.1 | 1.2 | 2.2 | 1.3 | 2.3 | 1.3 |
| **3b. I wait until I am sure a problem with my health is serious.** | 2.5 | 1.3 | 2.6 | 1.4 | 2.6 | 1.3 | 2.3 | 1.3 | 2.5 | 1.4 |
| **3c. I take action immediately when I have a health problem** | 2.3 | 1.3 | 2.5 | 1.3 | 2.4 | 1.3 | 2.2 | 1.2 | 2.1 | 1.2 |
| **3d. A problem with my health will go away on its own.** | 1.7 | 1.1 | 1.8 | 1.2 | 1.7 | 1.1 | 1.5 | 0.9 | 1.7 | 1.1 |
| **Subscale 4: Restrictive Emotionality** | 2.9 | 0.6 | 2.9 | 0.6 | 2.9 | 0.6 | 2.8 | 0.6 | 2.8 | 0.6 |
| **4a. It is important to talk to others about my feelings.** | 2.8 | 1.2 | 3.0 | 1.2 | 2.8 | 1.3 | 2.9 | 1.2 | 2.8 | 1.2 |
| **4b. I believe in remaining strong at all times** | 4.0 | 1.1 | 3.9 | 1.1 | 4.1 | 1.1 | 3.8 | 1.1 | 3.9 | 1.0 |
| **4c. Acknowledging my own emotions is always helpful.** | 2.1 | 1.1 | 2.3 | 1.1 | 2.1 | 1.2 | 2.0 | 1.0 | 2.0 | 1.0 |
| **4d. Physical pain in my body would never lead me to show any emotions** | 2.5 | 1.3 | 2.4 | 1.2 | 2.6 | 1.4 | 2.4 | 1.2 | 2.5 | 1.3 |
| **Subscale 5: Fear of Being Perceived as Gay** | 2.2 | 0.7 | 2.3 | 0.8 | 2.3 | 0.7 | 2.1 | 0.8 | 2 | 0.7 |
| **5a. Having medical exams below the waist would disrespect my sexuality.** | 1.4 | 1.0 | 1.4 | 1.0 | 1.5 | 1.0 | 1.4 | 1.0 | 1.3 | 1.0 |
| **5b. Medical professionals touching me below the waist is fine when it relates to my health.** | 1.7 | 1.2 | 1.8 | 1.2 | 1.8 | 1.1 | 1.8 | 1.2 | 1.7 | 1.1 |
| **5c. Health problems below the waist would be embarrassing to me.** | 2.1 | 1.3 | 2.2 | 1.3 | 2.3 | 1.3 | 1.8 | 1.1 | 1.9 | 1.2 |
| **5d. I would be comfortable with others knowing about health problems below the waist.** | 3.4 | 1.4 | 3.7 | 1.4 | 3.4 | 1.4 | 3.3 | 1.4 | 3.1 | 1.5 |
| **Subscale 6: Medical Mistrust** | 2.3 | 0.8 | 2.3 | 0.8 | 2.4 | 0.8 | 2 | 0.7 | 2.3 | 0 |
| **6a. Medical professionals want men of my race to live a long time.** | 2.5 | 1.4 | 2.4 | 1.3 | 2.6 | 1.3 | 2.6 | 1.5 | 2.5 | 1.4 |
| **6b. Men of my race are treated like guinea pigs by medical professionals.** | 1.7 | 1.1 | 1.6 | 1.1 | 1.8 | 1.1 | 1.4 | 0.8 | 1.8 | 1.2 |
| **6c. Men of my race trust medical professionals.** | 2.6 | 1.2 | 2.9 | 1.3 | 2.6 | 1.2 | 2.3 | 1.2 | 2.3 | 1.1 |
| **6d. Men of my race rarely receive quality medical care** | 2.4 | 1.4 | 2.4 | 1.3 | 2.6 | 1.4 | 1.7 | 1.2 | 2.5 | 1.4 |

**Abbreviations:** STD, Standard Deviation.
